# Clonal relationships of memory B cell subsets in autoimmune mice

**DOI:** 10.3389/fimmu.2023.1129234

**Published:** 2023-03-01

**Authors:** Alaitz Aranburu, Erik Engström, Natalija Gerasimcik, Samuel Alsén, Alessandro Camponeschi, Ulf Yrlid, Ola Grimsholm, Inga-Lill Mårtensson

**Affiliations:** ^1^ Department of Rheumatology and Inflammation Research, Institute of Medicine, Sahlgrenska Academy, University of Gothenburg, Gothenburg, Sweden; ^2^ Department of Microbiology and Immunology, Institute of Biomedicine, Sahlgrenska Academy, University of Gothenburg, Gothenburg, Sweden; ^3^ Sahlgrenska Cancer Center, Department of Surgery, Institute of Clinical Sciences, University of Gothenburg and Sahlgrenska University Hospital, Gothenburg, Sweden; ^4^ Department of Pathophysiology and Allergy Research, Center for Pathophysiology, Infectiology and Immunology, Medical University of Vienna, Vienna, Austria

**Keywords:** memory B cell (MBC), Lineage tree analysis, Autoreactivity, H-CDR3, autoimmune disease

## Abstract

Immunological memory protects our body from re-infection and it is composed of a cellular and a humoral arm. The B-cell branch with its memory B cells (MBCs), plasma cells and antibodies, formed either in a germinal centre (GC) -dependent or -independent manner, ensure that we can rapidly mount a recall immune response. Previous work in immunised wildtype (WT) mice have identified several subsets of MBCs whereas less is known under autoimmune conditions. Here, we have investigated the heterogeneity of the MBC compartment in autoimmune mouse models and examined the clonal relationships between MBC subsets and GC B cells in one of the models. We demonstrate the presence of at least four different MBC subsets based on their differential expression pattern of CD73, CD80 and PD-L2 in surrogate light chain-deficient (SLC^-/-^), *MRL^+/+^* and MRL^lpr/lpr^ mice, where most of the MBCs express IgM. Likewise, four MBC subsets could be identified in WT immunised mice. In SLC^-/-^ mice, high-throughput sequencing of Ig heavy chains demonstrates that the two CD73-positive subsets are generally more mutated. Lineage tree analyses on expanded clones show overlaps between all MBC subsets and GC B cells primarily in the IgM sequences. Moreover, each of the three IgM MBC subsets could be found both as ancestor and progeny to GC B cells. This was also observed in the IgG sequences except for the CD73-negative subset. Thus, our findings demonstrate that several MBC subsets are present in autoimmune and WT mice. In SLC^-/-^ mice, these MBC subsets are clonally related to each other and to GC B cells. Our results also indicate that different MBC subsets can seed the GC reaction.

## Introduction

Memory B cells (MBCs) are responsible for life-long immunity to pathogens encountered earlier in life and are together with long-lived plasma cells (PCs) crucial for the maintenance of serological memory. It has become evident that in response to foreign antigens MBCs can be divided into several subsets in both mice and humans (1–4). MBCs can derive from germinal centre (GC)-dependent and -independent responses (5–7). GC formation leads to clonal expansion of antigen-activated B cells after which they take on a fate as MBCs or long-lived plasma cells (PCs). In the GC, V_H_ gene segments undergo diversification by introduction of somatic mutations. MBCs can be of IgM and switched isotype, e.g., IgG, and in humans, the CD27 marker has for many years been used to distinguish MBCs from naïve B cells (8, 9). However, CD27 is not a marker for MBCs in mice. Not until relatively recently have a set of markers - CD73, CD80, PD-L2 - been identified that can distinguish MBCs from naïve B cells in mice (10). These markers have been used to examine typical T-cell dependent immune responses, e.g., against the hapten NP or sheep red blood cells (SRBC) (10, 11). More recently, it has been suggested that the different subsets have different functions where CD80^+^PD-L2^+^ MBCs rapidly differentiate into antibody-forming cells whereas their double negative counterpart would seed the germinal centre (GC) reaction, independent of isotype (10, 12). Others have suggested that IgM^+^ MBCs seed the GCs whereas the IgG^+^ cells differentiate into PCs (11). Additionally, it has been shown that CD73^+^ MBCs are more mutated than their negative counterpart (6). It is also becoming increasingly clear that both extrafollicular (EF) and GC reactions give rise to somatically mutated antibodies (5). Altogether, experiments conducted on immunised mice support the notion that there are subsets of MBCs with distinct characteristics and roles during the MBC response.

One of the hallmarks of autoimmune diseases is the production of autoantibodies that typically arise during T-cell dependent immune reactions. This also include immunological memory, analogous to immune responses against foreign antigens. In autoimmune diseases though, the formation of MBCs is a predicament, as MBCs that are formed in response to autoantigens would retain life-long autoimmunity. Autoantibodies have been the subject of extensive research showing clonal expansion of autoreactive B cells already in the late 1980s (13). However, different MBC subsets have not been extensively studied in autoimmune mouse models. Nevertheless, expansion of an autoreactive MBC subset defined as CD21^-/low^ has been described in the lupus-prone mouse model NZB/W (14), a subset also termed age-associated B cells (ABCs) (15). Furthermore, we have shown that there is an expansion of ABCs also in surrogate light chain deficient (SLC^-/-^) mice and in lupus-prone *MRL^lpr/lpr^* mice (16, 17). In addition, some of these ABCs express CD73 and CD80. We also found that ABCs in SLC^-/-^ mice are autoreactive and that their Ig heavy chains form clonal trees. SLC^-/-^ mice represent a model of autoimmunity with spontaneous T-cell dependent autoimmune reactions, including the formation of GCs and elevated serum levels of typical lupus autoantibodies, as a result of defective selection of Ig heavy chains during B-cell development (17–19). Moreover, CD73^+^CD80^+^PD-L2^+^ B cells are present in the spleen of SLC^-/-^ mice, of which some have undergone class switch recombination (CSR), and IgM^+^ and IgG^+^ cells show signs of somatic hypermutation (SHM) (18), hence typical of MBCs. However, the CD73, CD80 and PD-L2 markers have not, to our knowledge, been extensively investigated in autoimmune mouse models to determine whether similar or equivalent subsets to those in WT immunised mice exist. Here using these markers, we have examined the presence of MBC subsets in autoimmune *MRL^+/+^*, *MRL^lpr/lpr^* and SLC^-/-^ mice in more detail and, in the latter, determined their clonal relationships to each other and to the GC B cells using deep sequencing of the Ig heavy chain.

## Results

### Spontaneous formation of several MBC subsets in SLC^-/-^ mice

**Figure 1:**
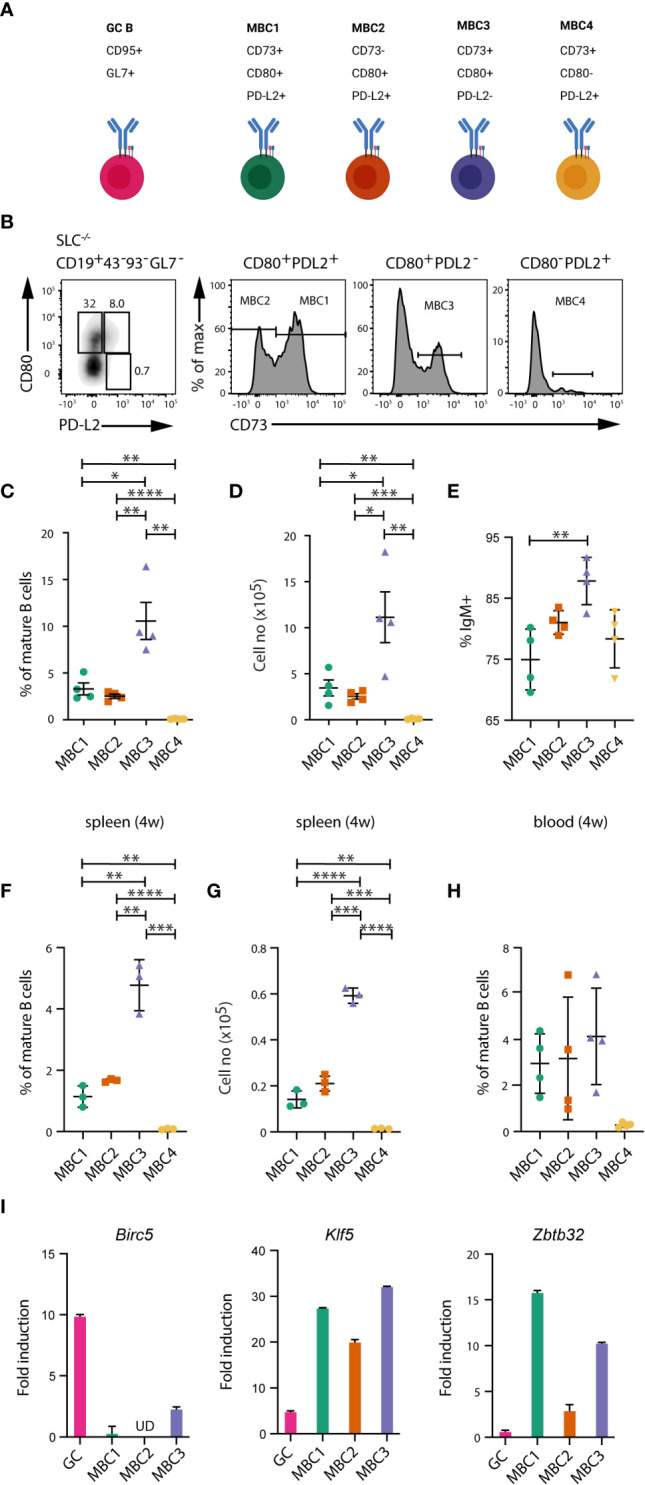
Several subsets of memory B cells in autoimmune mice. **(A)** Schematic overview of MBC subsets and their development in SLC^-/-^ mice. Created with Biorender.com. **(B–E)** Flow cytometry analysis of spleen cells and peripheral blood leukocytes from SLC^-/-^ mice. **(B)** FACS plots show the different MBC subsets. Cells are gated on CD19^+^CD43^-^CD93^-^GL7^-^ mature B cells and then using CD73, CD80 and PD-L2 for detection of MBCs. **(C, D)** Graph shows the proportions **(C)** and absolute numbers **(D)** of MBC1 (CD73^+^CD80^+^PD-L2^+^), MBC2 (CD73^-^CD80^+^PD-L2^+^), MBC3 (CD73^+^CD80^+^PD-L2^-^) and MBC4 (CD73^+^CD80^-^PD-L2^+^). **(E)** Graph shows the proportions of IgM-expressing cells within MBC1-4 subsets. **(F, G)** Graph shows the proportions **(F)** and absolute numbers **(G)** of MBC1-4 in four weeks old SLC^-/-^ mice. **(H)** Flow cytometry analysis of peripheral blood leukocytes from SLC^-/-^ mice. Graph shows the different MBC subsets. Cells are gated as in **(B)**. **(I)** QPCR analysis of *Birc5*, *Klf5* and *Zbtb32* mRNA levels in GC B cells and MBC subsets from SLC^-/-^ mice, with b-actin as internal ctrl. Fold induction was calculated using a pool of FO B cells from WT mice (n=3) as reference (fold induction=1). Results in **(B-F)** are representative and from at least three independent experiments with n=4-6 in each experiment. Results in **(G, H)** are representative from two independent experiments with n=4-5 in each experiment. Results in **(I)** from two independent pools of 5-6 mice. *P < 0.05; **P < 0.01; ***P < 0.001; ****P < 0.0001.

Our previous data have indicated the presence of several MBC subsets in the autoimmune SLC^-/-^ mouse model (18, 20). Here, we set out to examine more thoroughly the presence of different MBC subsets in spleen and peripheral blood. Using flow cytometry, we observed three main subsets termed MBC1 (CD73^+^CD80^+^PD-L2^+^), MBC2 (CD73^-^CD80^+^PD-L2^+^) and MBC3 (CD73^+^CD80^+^PD-L2^-^) (**Figures 1A, B**). A minor subset of CD73^+^CD80^-^PD-L2^+^ termed MBC4 was also observed. Within the pool of mature B cells in the spleen, MBC1 and 2 each represent 2-3% and MBC3 almost 10% whereas MBC4 was <0.1% at an age of 20-24 weeks (**Figure 1C**). In cell numbers, this corresponds to approximately 2-3 x10^5^ cells for MBC1 and 2, a million for MBC3 and 10 x10^3^ for MBC4 (**Figure 1D**). All four subsets expressed mostly the IgM isotype, with MBC1 representing the subset with least (75%), and thus with most switched cells (**Figure 1E**). In addition, examining the spleen of mice at an age of four weeks we discovered that MBC1-4 are present also in these young mice albeit numbers are lower than later in life (**Figures 1F, G**). In these same mice we could also detect these MBC subsets in the peripheral blood (**Figure 1H**), indicating that they also recirculate in the blood. Moreover, we found the four subsets in lymph nodes where we also could detect GC B cells (**Supporting Information 1**). Taken together, these data show that MBC3 is the most abundant subset, regardless of age or organ type (spleen or lymph nodes), while MBC4 is barely detectable.

Next, we validated the MBC phenotype by qPCR on a few genes that have been previously described in immunised wildtype (WT) mice (12), including *Zbtb32* that is highly expressed in MBCs. We re-analysed this dataset (12), where we identified *Klf5* to be one of the highly expressed genes in MBCs (not shown). Our qPCR analysis confirmed expression of both factors in MBC1-3 from SLC^-/-^ mice (**Figure 1I**). Due to the very low numbers of MBC4, these were not analysed. We also observed that the expression of *Zbtb32* was lower in MBC2 as compared to the two other MBC subsets. Expression of *Birc5* was used as comparison since it is normally highly expressed in GC B cells (21), which was the case also in the spontaneously forming GC B cells from SLC^-/-^ mice. These results support the notion that several MBC subsets can be phenotypically distinguished also under autoimmune conditions.

### Similar distribution of MBC subsets also in MRL^+/+^, MRL^lpr/lpr^ and WT immunised mice

**Figure 2:**
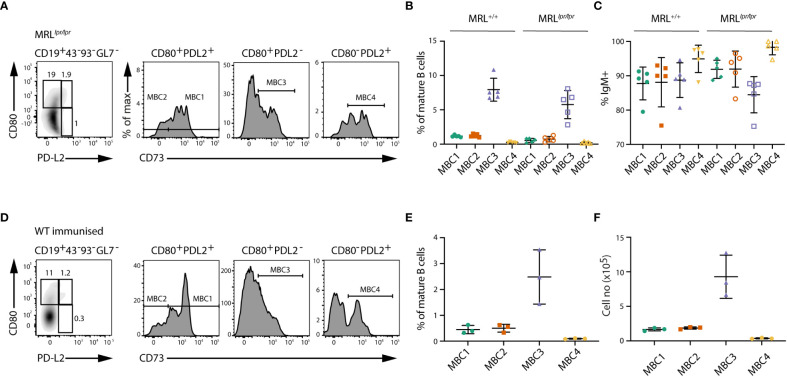
Phenotypic characterisation of MBC subsets in MRL^lpr/lpr^ mice and WT immunised mice. **(A)** FACS plots show the different MBC subsets in MRL^lpr/lpr^ mice. Cells are gated on CD19^+^CD43^-^CD93^-^GL7^-^ mature B cells and then using CD73, CD80 and PD-L2 for detection of MBCs as in Figure 1. **(B)** Graph shows the proportions of MBC1-4 in MRL^+/+^ and MRL^lpr/lpr^ mice. **(C)** Graph shows the proportions of IgM-expressing cells within MBC1-4 subsets in MRL^+/+^ and MRL^lpr/lpr^ mice. **(D)** FACS plots show the different MBC subsets in SRBC-immunised WT mice five months after booster dose. **(E, F)** Graph shows the proportions **(E)** and absolute numbers **(F)** of MBC1-4 in SRBC-immunised WT mice five months after booster. Results in **(A–C)** are representative from two independent experiments with n=4-5 in each experiment. Results in **(D–F)** are representative from two independent experiments with n=3 in each experiment.

We then analysed *MRL^+/+^* and *MRL^lpr/lpr^* mice to compare our results on the distribution of MBC subsets in SLC^-/-^ mice to other autoimmune models. The *MRL^+/+^* model has been shown to display autoimmune characteristics including GC formation and in the presence of the *lpr* mutation the mice develop lupus-like disease (22). We have previously demonstrated that some of the ABCs developing in both these models express CD73 and CD80. Here we show the presence of MBC1-4 also in these mice, and that MBC3 is the most abundant subset (**Figures 2A, B**). Also, in *MRL^+/+^* and *MRL^lpr/lpr^* mice IgM is the dominant isotype in all MBC subsets (**Figure 2C**).

We additionally analysed SRBC-immunised WT mice five months after booster dose. In these mice we could identify the same four MBC subsets based on surface phenotype, with the most abundant being MBC3 (**Figures 2D–F**). Although the proportions of MBC1-4 were lower in immunised WT mice than in SLC^-/-^ mice, due to the low number of splenic B cells in SLC^-/-^ mice, the actual cell numbers were rather similar.

All in all, these data suggest that in autoimmune mice, i.e., *SLC^-/-^*, *MRL^+/+^* and *MRL^lpr/lpr^*, at least four subsets of MBCs develop. The proportion of the four subsets is consistently similar, with MBC3 being the largest and MBC4 the smallest, regardless of whether they develop spontaneously as seen in autoimmune models or after immunisation of WT mice with a particulate antigen.

### Differences in mutation frequencies between MBC subsets

The presence of at least four MBC subsets in SLC^-/-^ mice led us to investigate the clonal relationships between MBC1-3 (due to the low number of MBC4, these were not analysed) and their relation to GC B cells, by determining their respective Ig heavy chain repertoire. To this end, we first investigated the expression of different V_H_ families in search for similarities and differences among the MBC subsets, using naïve and GC B cells for comparison. The data derived from NGS resulted in 14138 unique IgM and 4505 unique IgG sequences. In both the IgM and IgG sequences from the three MBC subsets, mainly three families were used, V_H_1, V_H_5 and V_H_14 though at varying proportions (**Supporting Information 2**).

**Figure 3:**
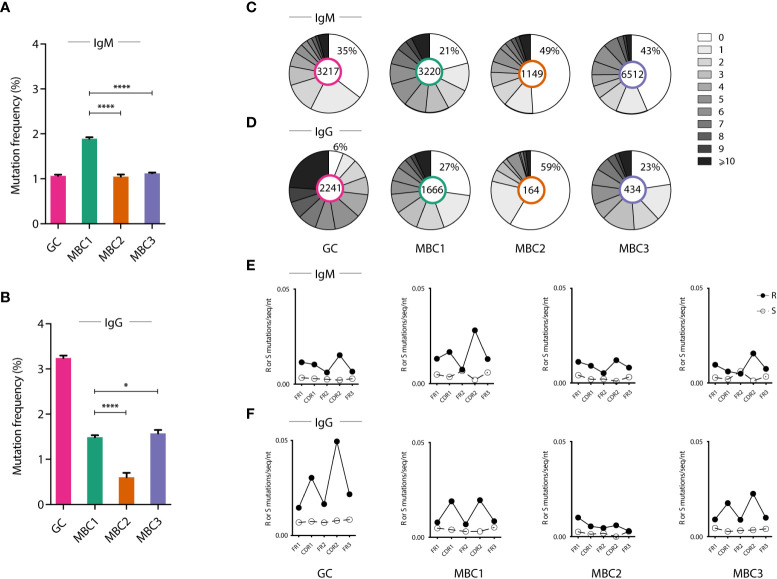
Mutation frequency in the different MBC subsets. **(A–F)** Ig heavy chain sequencing analysis from sorted GC B cells and MBC subsets. **(A, B)** Graphs show the frequency of somatic mutations in indicated cell populations in IgM **(A)** and IgG **(B)** expressing cells. **(C, D)** Pie charts show the number of mutations in indicated cell populations in IgM **(C)** and IgG **(D)** expressing cells. **(E, F)** Graphs show the mutation pattern with replacement (R) and silent (S) mutations along the Ig molecule (framework regions and CDRs) in IgM **(E)** and in IgG **(F)**. Results from a pool of 6 mice. *P < 0.05; ****P < 0.0001.

To determine whether the three MBC subsets showed signs of SHM we calculated the mutation frequency and the range of mutations in the expressed V_H_ genes independent of family, using GC B cells for comparison. GC B cells and the MBC1 subset displayed signs of SHM, consistent with our previous results (18), and so did MBC2 and MBC3 (**Figures 3A–D**). Assessing the isotypes separately we observed that IgM sequences from MBC1 showed the highest mutation frequency and highest proportion of sequences with mutations. In the IgG fraction, however, these attributes were linked to the GC B cells although mutations were observed also in the MBC subsets with the least in MBC2. Analysing these same parameters in the different V_H_ families (V_H_1, V_H_5 and V_H_14), most noticeable was that V_H_14 was often more mutated than the other two families in both IgM- and IgG-expressing MBCs (**Supporting Information 3**). Next, we analysed the sequences for mutations in complementarity determining region (CDR)1 and CDR2 and FRs (framework regions). This showed that the frequency of replacement mutations was increased in the CDRs, in both IgM and IgG sequences, in all subsets except MBC2 where it was not as evident and especially not in the IgG sequences (**Figures 3E, F**). Taken together, these results support that the MBC1-3 subsets in autoimmune SLC^-/-^ mice are indeed MBCs. Moreover, the MBC2 subset, lacking CD73 expression, and especially the IgG sequences, is overall less mutated than the CD73-expressing subsets, which is similar to observations in immunised WT mice (6).

### Clonal expansion of GC B cells and MBCs

**Figure 4:**
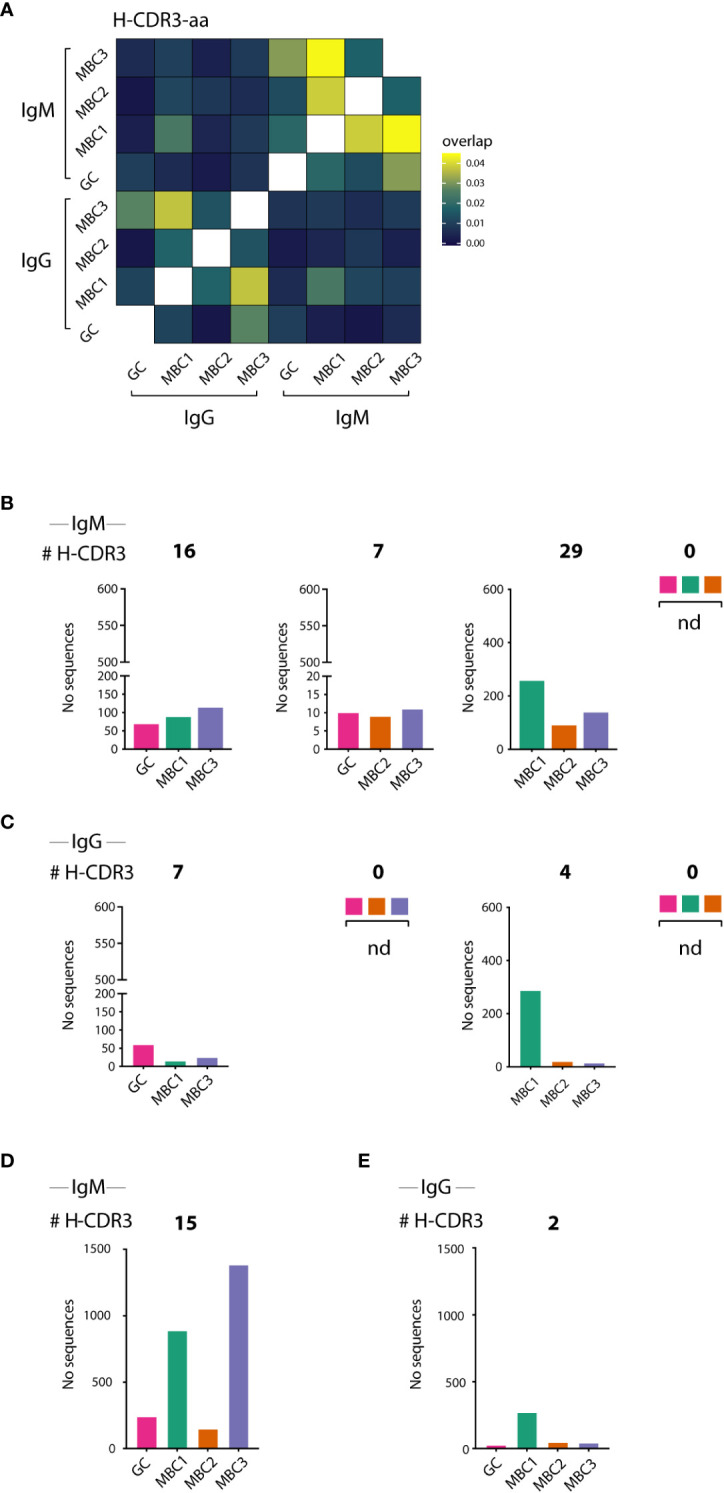
H-CDR3 sharing between GC B cells and MBC subsets. **(A–E)** H-CDR3 sharing analysis of VDJH sequences in sorted IgM- and IgG-expressing GC B cells and MBC subsets. **(A)** Heatmap shows sharing between IgM and IgG-expressing GC B cells and MBC subsets. **(B–E)** Graphs show the number of shared H-CDR3s between three **(B, D)** and four **(C, E)** populations for IgM and IgG sequences as indicated. Results from a pool of 6 mice.

Next, to delineate possible clonal relationships between the GC B cells and the three MBC subsets we analysed the sequences for shared clones, defined as those having 100% amino acid identity in the H-CDR3. In the IgM H-CDR3 repertoire 43% of all sequences were shared between at least two subsets, which corresponded to 7% of the H-CDR3 clones (**Table 1**). The extent of sharing in the IgG H-CDR3 repertoire was similar (8%) to that of IgM, however, fewer (28%) sequences were shared overall. Generating a heatmap where all MBC subsets and GC B cells as well as the two isotypes were analysed together, we observed that sharing of H-CDR3 differed between isotypes: there was substantial sharing between MBCs in the IgM fraction, whereas in the IgG fraction it was modest and most evident between MBC1 and MBC3 (**Figure 4A**), the two subsets with more mutations. We also observed sharing between GC and MBC subsets, especially with MBC3 and MBC1. Sharing between IgM and IgG was weak and was mainly observed for MBC1. Analysis of IgM and IgG sequences separately confirmed the data in the heatmap (**Supporting Information 4A, B**).

**Table 1:** Summary of unique H-CDR3 clone and sequences.

All IgM	GC	MBC1	MBC2	MBC3	Total
# Clones	1 734	921	697	3 595	6 409
# Seq	3 217	3 220	1 189	6 512	14 138
All IgG	GC	MBC1	MBC2	MBC3	Total
# Clones	1 267	739	96	260	2 362
# Seq	2 241	1 666	164	434	4 505
Single IgM	GC	MBC1	MBC2	MBC3	(%)
# Clones	1 536	646	573	3 212	93
# Seq	2 407	954	708	4 021	57
Shared IgM	GC	MBC1	MBC2	MBC3	(%)
# Clones	198	275	124	383	**7**
# Seq	810	2 266	481	2 491	**43**
Shared IgM^a^	GC	MBC1	MBC2	MBC3	Total/%
# Seq	240	889	149	1385	2663/19
Single IgG	GC	MBC1	MBC2	MBC3	(%)
# Clones	1 215	677	78	193	92
# Seq	1 936	974	83	240	72
Shared IgG	GC	MBC1	MBC2	MBC3	(%)
# Clones	50	62	18	66	**8**
# Seq	302	690	81	192	**28**
Shared IgG^b^	GC	MBC1	MBC2	MBC3	Total/%
# Seq	27	272	47	43	389/9

a Shared IgM sequences by GC B cells and the three MBC subsets as shown in **Figure 5**.

b Shared IgG sequences by GC B cells and the three MBC subsets as shown in **Figure 5**.

The number of unique H-CDR3 clone and sequences (Seq) before clonotype analysis are referred as “all”; After clonotype analysis, H-CDR3 clones NOT shared (Single), and corresponding sequences, as well as H-CDR3 in common (Shared), and corresponding sequences, are shown. Data corresponds to IgM or IgG sequences only.

Next, we divided clones sharing H-CDR3 into those containing three or all four subsets that expressed IgM (**Figures 4B, D**) or IgG (**Figures 4C, E**). First, we examined the H-CDR3 clones shared by three subsets, and although not all potential combinations were found, GC-MBC1-MBC3 (16 clones) and MBC1-MBC2-MBC3 (29 clones) were the most abundant among the IgM as well as IgG sequences, the latter represented by 7 and 4 clones, respectively (**Figures 4B, C**). Among those that contained all four subsets were 15 clones expressing IgM, and remarkably these contained 19% of the sequences whereas only two clones were observed among the IgG sequences (**Figures 4D, E**; **Table 1**), demonstrating substantial expansion of IgM clones containing all MBC subsets as well as GC B cells. We conclude that GC B cells and all the MBC subsets are clonally related albeit the composition of the different clones, i.e., number of sequences contributed by each cell population and isotype varies.

### Increased levels of SHM in shared H-CDR3 clones in the IgM repertoire

Our observations on the high (2663) number and proportion (19%) of sequences assigned to the expanded H-CDR3 clones that were shared between all four populations (**Table 1**; **Figure 5A**), primarily in the IgM fraction, prompted us to study further SHM in these sequences. These were first compared to those from not shared H-CDR3 clones, expanded or not, herein termed ‘single’. To this end, we calculated the mutation frequency in IgM and IgG sequences derived from shared and single H-CDR3 clones. In the IgM fraction, we found that the mutation frequency was highest when a given population was present in shared as opposed to ‘single’ H-CDR3 clones (**Figure 5B**). The increase in mutation frequency was most pronounced in MBC3 and significant for all four populations. We next looked at the range of mutations from single and shared H-CDR3 clones. Again, the shared H-CDR3 clones contained more mutated IgM sequences which was true for all four populations (**Figure 5C**). Analysis of the IgG sequences revealed an almost opposite pattern, especially GC B cells and MBC1, where those belonging to single H-CDR3 clones were more mutated than those that were shared (**Figures 5D, E**). In summary, we show that B cells that are part of shared H-CDR3 clones have been subjected to more SHM in the IgM, but not in the IgG repertoire.

**Figure 5:**
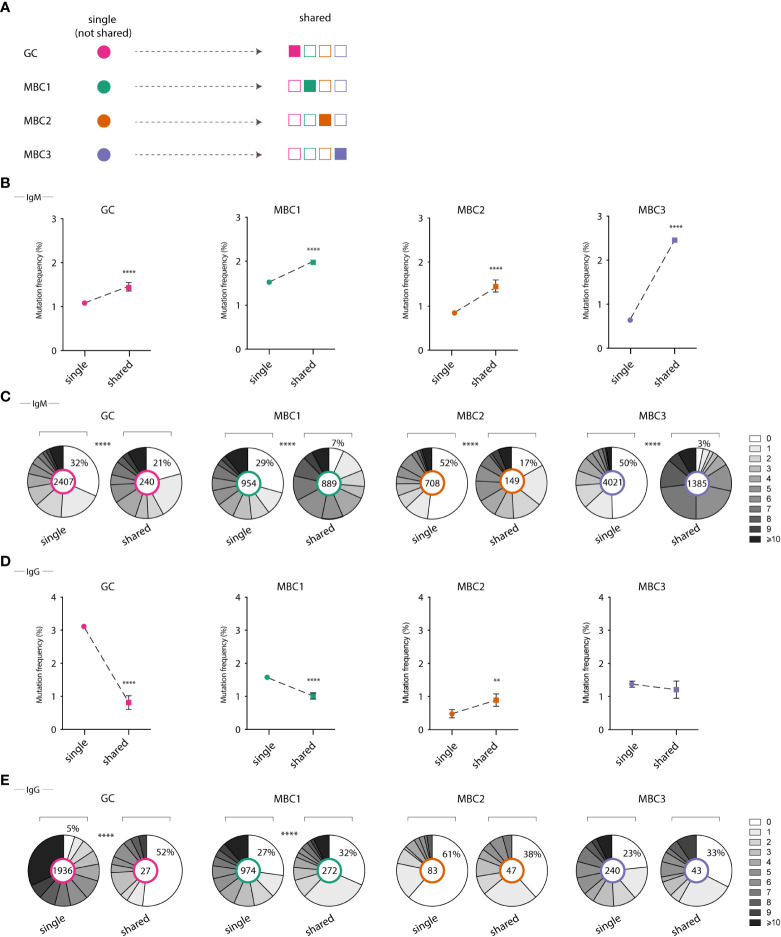
Mutation frequency in single vs shared clones in GC B cells and MBC subsets. **(A)** Illustrative graph shows definition of single and shared H-CDR3s **(B, D)** Graphs show mutation frequency in single and shared H-CDR3s for IgM sequences **(B)** and IgG sequences **(D)**. **(C, E)** Pie chart shows the number of mutations in single vs shared H-CDR3s for IgM sequences **(C)** and IgG sequences **(E)**. Results from a pool of 6 mice. **P<0.01; ****P<0.0001.

### Lineage tree analyses show clonal relationships between MBC subsets and GC B cells

**Figure 6:**
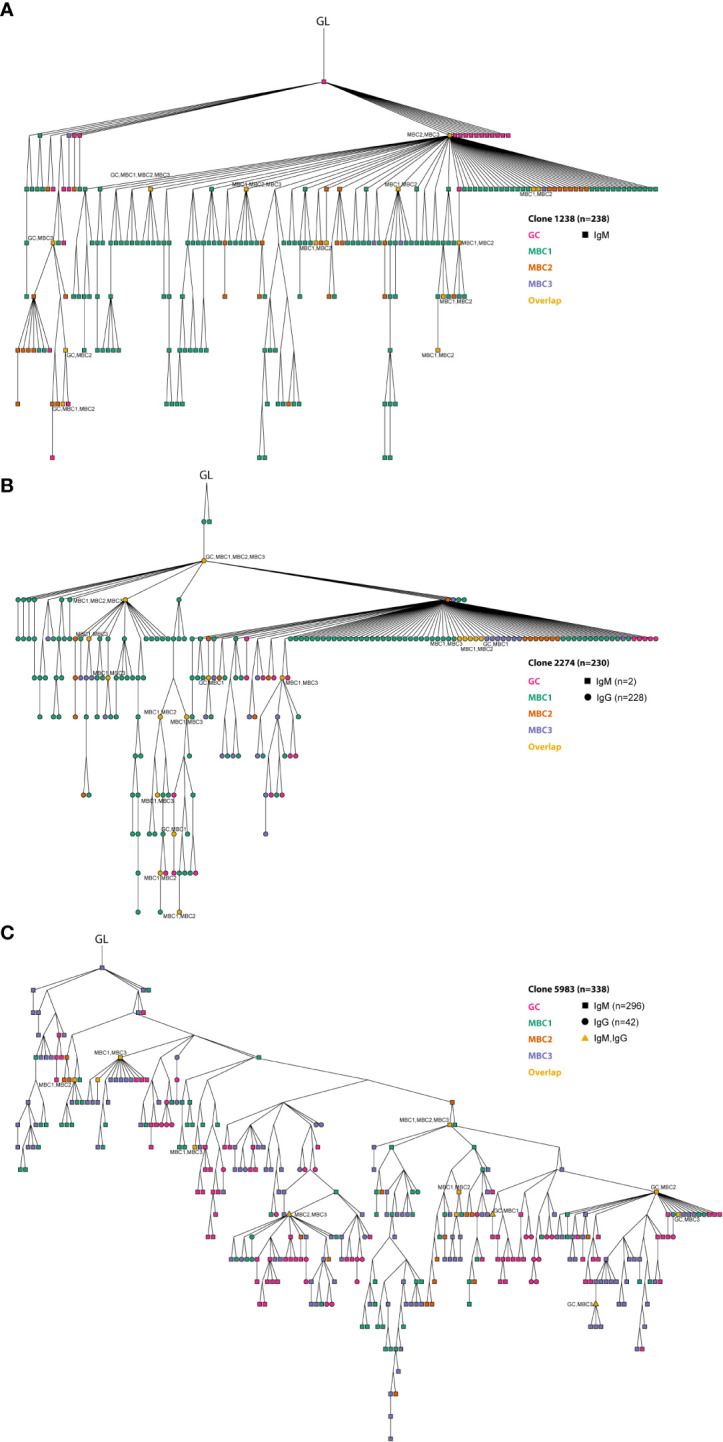
Clonal relationships between GC B cells and MBC subsets. **(A–C)** Three expanded clones are shown from the lineage tree analysis. Germline (GL) is indicated in the figure. The different MBC subsets and GC B cells are indicated in the figure. At certain nodes overlap between different MBC subsets and with GC B cells can be observed (in orange). Overlapping sequences for IgM and IgG are also indicated (triangle shaped). Results from a pool of 6 mice. Overlapping nodes without text are identical to those of their ancestor.

**Figure 7:**
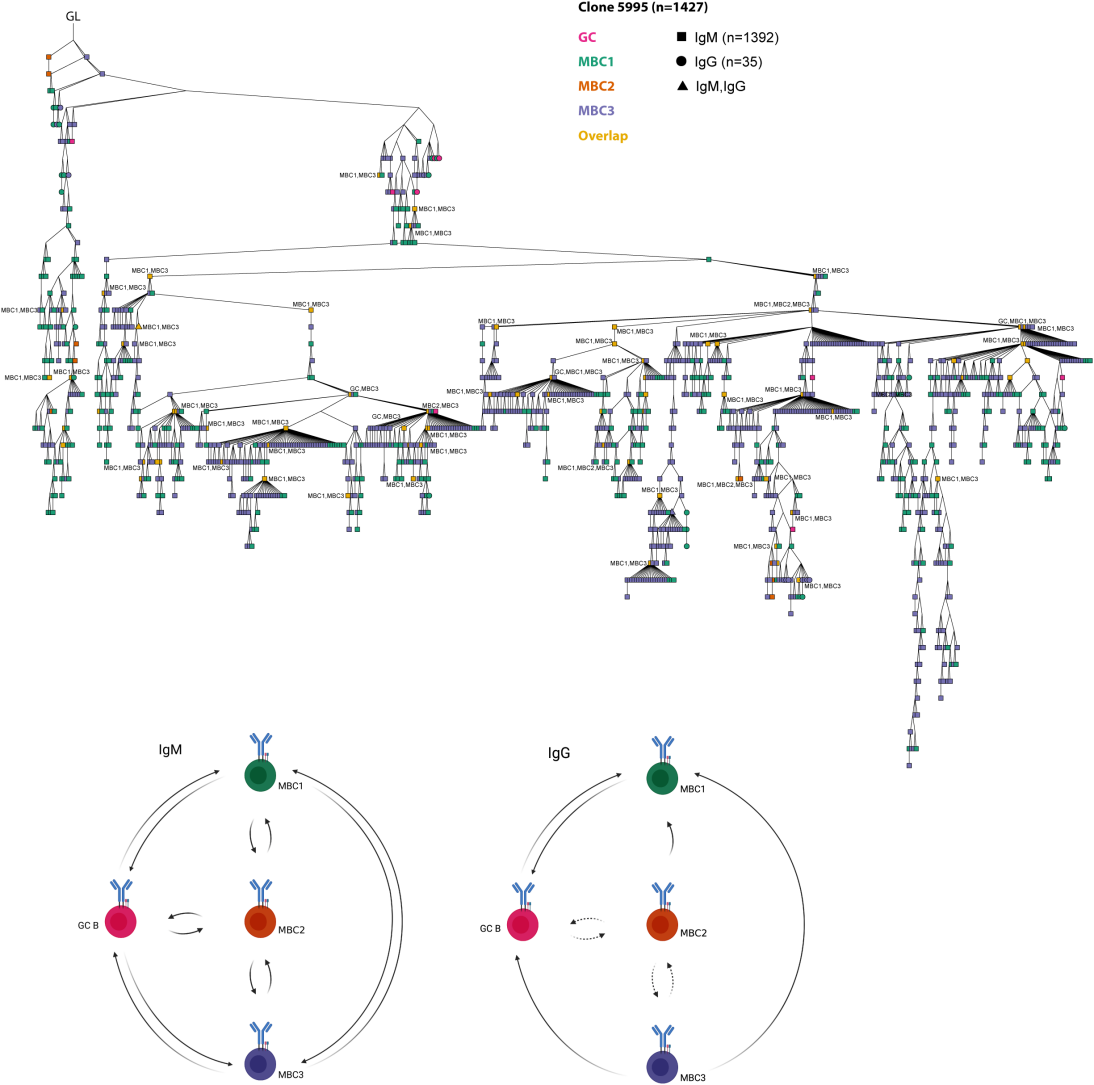
A model on clonal relationships between MBC subsets and GC B cells. **(A)** The largest clone from the lineage tree analysis. GL is indicated in the figure. The different MBC subsets and GC B cells are indicated in the figure. At certain nodes overlap between different MBC subsets and with GC B cells can be observed (in orange). Overlapping sequences for IgM and IgG are also indicated (triangle shape). Overlapping nodes without text are identical to those of their ancestor. Results from a pool of 6 mice. **(B)** A model on the clonal relationships between MBC1-3 and GC B cells shown for IgM and IgG separately based on our lineage tree analyses. Created with Biorender.com.

Thereafter, we performed lineage tree analyses based on sharing of the H-CDR3 (equal or greater than 85% identity at the amino acid level) to investigate clonal relationships between the GC B cells and the three MBC subsets. To this end, we initially focused on three clones that were among the top 10 most expanded clones after combining sequences from GC B cells and MBCs from both isotypes. The respective germline (GL) sequence was inferred from IMGT. Clone 1238 (n=238) contained only IgM sequences, clone 2274 (n=230) only IgG except for two IgM sequences, clones 5983 (n=338) and 5995 (n=1427) contained both isotypes (**Figures 6A–C**, **7A**). These four clones contained sequences from all populations. Some of the sequences from one population overlapped (orange nodes) with that of another, i.e., they shared the same sequence. In fact, at some nodes, at least three of the populations overlapped, for instance GC B cells and two of the MBC subsets or the three MBC subsets, which we observed for both IgM and IgG sequences (**Figures 6A–C**, **7A**). In addition, we observed isotype switching from e.g., IgM GC B cells to IgG MBC1 and IgM MBC3 to IgG GC B cells, but not all possible combinations (**Figures 6A–C**, **7A**), which would be consistent with more limited overlap between IgM and IgG H-CDR3 (**Figure 4A**). We also noticed that the trees contained fewer sequences from the MBC2 subset (**Figures 6A–C**, **7A**), which is consistent with the H-CDR3 sharing between subsets (**Figure 4A**). Studying the hierarchy of the trees we observed that each of the three MBC subsets could be found as a progeny to GC B cells, and vice versa albeit some combinations were less frequent (**Figures 6A–C**, **7A**; **Supporting Information 5**). Furthermore, any of the three MBC subsets could be found as an ancestor node to any of the other MBC subsets and to GC B cells. Similar results were obtained when we analysed the remainder of the top 10 most expanded clones; two of which were composed mainly of IgG-expressing GC B cells and the others a mixture of all subsets (**Supporting Information 5**). Taken together, this strongly suggests that the MBC subsets are clonally related to each other and to the GC B cells.

## Discussion

The use of CD73, CD80 and PD-L2 has significantly advanced our understanding of mouse MBCs separating them into subsets as well as demonstrating how different MBCs give rise to PCs and seed new GCs during secondary responses (5). There seem to be a gradient of maturity among the different subsets where CD73^+^CD80^+^PD-L2^+^ are most mature and this is in line with their capacity to form antibody-secreting cells, whereas CD80^-^PD-L2^-^ (CD73^+/-^), which are also much less mutated, may re-enter the GC upon secondary infection/immunisation (12). Recent data is also pointing towards this direction where mainly naïve B cells or IgM MBCs are recruited into recall responses whereas IgG cells are directed towards a PC fate (23–25). In mice immunised with NP-CGG the mutation frequency of these different MBC subsets varies where CD80^-^PD-L2^-^ are the least and, CD80^+^PD-L2^+^ the most, mutated with IgM being less mutated than IgG (10). However, to our knowledge, the clonal relationships between these different subsets have not been studied in detail. In our in-depth analysis of the clonal relationships between MBC1-3 we can now show that at least in the SLC^-/-^ mice they are clonally related thereby pointing towards a common clonal origin. The fact that different subsets are clonally related does not exclude that they may have different functional capacities.

Here we phenotypically identified the same MBC subsets in autoimmune mice as those in immunised WT mice showing similar proportions of IgM and switched cells. That they are different MBC subsets also in an autoimmune setting (SLC^-/-^) is supported by the expression of for instance *Zbtb32* with the lowest levels in MBC2. This gene has been previously shown to be highly expressed by MBCs and to be a negative regulator of antibody recall responses (26). Our analyses also demonstrate that the CD73-lacking MBC2 cells have fewer mutations, thus, further supporting that this subset differs from the other two, and that the lack of CD73 is associated with lower mutation frequency also in autoimmune mice, as previously demonstrated in immunisation models (6). Whether the lower mutation frequency in MBC2 indicates that these cells leave the GC reaction early and differentiate into PCs is unknown but a possibility especially in light of their low levels of *Zbtb32*, as this factor is involved in the attenuation of the Ab production during recall responses (26). Alternatively, MBC2s are to some extent GC-independent (6, 7, 27). CD73 expression is tightly modulated during B cell responses, increasing within the GC environment on B and T_FH_ cells over time, contributing to the CD73 enzymatic activity in the GC (28). In this respect, it is of interest that SLC^-/-^ mice have disproportionally high numbers of T_FH_ cells in their GCs (18), potentially favoring MBC1 and MBC3. These two subsets appear to be more similar, for instance expressing the highest levels of *Klf5* and *Zbtb32*,previously shown to be highly expressed in MBCs from WT immunised mice (12), displaying higher mutation frequencies than MBC2 as well as more extensive sharing of the H-CDR3 repertoire within each isotype. Nevertheless, whether there are even more MBC subsets than MBC1-4 in autoimmune mice is unclear. Previous work in Plasmodium infections have described a population of antigen-specific IgM^low^IgD^+^ MBCs that lack both CD73 and CD80 (29). However, if such a population is present in autoimmune models is more difficult to test, as the (auto)antigen is mostly unknown.

During our analyses we observed that, irrespective of subset, the mutation frequency in those sharing H-CDR3 is consistently higher than that in single sequences when it comes to the IgM isotype. The picture is slightly different in the IgG sequences, especially for GC and MBC1, that were less mutated in the shared compared to the single sequences. We do not know the reason behind this difference, but a possibility is that IgG MBC1 that are clonally related to the IgM MBCs and GCs exit the GC reaction early resulting in a lower mutation frequency. Nevertheless, our lineage tree analysis shows several notable features regarding the clonal relationships between MBC subsets and GC B cells. First, we noted that all MBC subsets could be found as progeny to any other MBC subset as well as to GC B cells. Second, the largest tree in our dataset, clone 5995, contained all MBC subsets and GC B cells that at certain nodes overlapped between two or more of these as well as between isotypes (**Figure 7A**). Third, in most trees, it was apparent that MBC2 was less frequent and also its sharing with for instance MBC3 more limited. Fourth, our data show that any of the IgM-expressing MBC subsets can be found as an ancestor node to a GC B cell, thus indicating that these MBCs could potentially seed the GC reaction (**Figure 7B**). With the same reasoning our results suggest that at least IgG-expressing MBC1 and MBC3 can also feed into the GC response.

CD73 is also expressed on human MBCs (30, 31), whereas the two other markers used herein, CD80 and PD-L2, are not (31, 32). However, it remains to be seen whether CD73-expressing human MBCs are more somatically mutated than those being CD73-negative, as in mice (6). Previously, human CD27^+^ MBCs have mainly been separated into subsets according to their isotype, i.e., IgD^+^ or IgD^-^ (9), but in recent years it has been shown that they also can be separated into different subsets based on CD27 levels, as well as other cell surface markers (30–33). In this regard, human CD27^+^ MBCs have been shown to consist of CD27^dull^ and CD27^bright^ subsets that are clonally related, irrespective of isotype (33). Moreover, several subsets have been inferred from their transcriptome in single-cell RNA-sequencing studies (34–36), as well as clonal relationships extensively examined between CD27^+^ IgM and switched MBCs (37–39). Clonal relationships between IgM and switched, as well as between switched isotypes have also been described in human immune-mediated diseases including autoimmune diseases (40). Although, it is unclear whether these clones derive from the same or different MBC subsets in the respective condition. In relation to our data it is of interest to note that we also observed overlaps between IgM and switched MBCs in addition to between MBC subsets.

In summary, our results demonstrate that the CD73, CD80 and PD-L2 markers, first identified in immunised WT mice, can distinguish different MBC subsets also in autoimmune mouse models, where the MBC3 subset is dominant. We also find that MBC2s, which lack CD73, display a lower mutation frequency in SLC^-/-^ mice, as previously observed in immunised WT mice (6). Furthermore, our lineage tree analyses in SLC^-/-^ mice show that all three MBC subsets are clonally related to each other and to GC B cells although MBCs lacking CD73 (MBC2) are less frequent. Whether the clonal relationships apply directly to other autoimmune settings is currently unclear and will have to await future studies.

## Materials and methods

### Mice

The SLC^-/-^ mice (41), previously backcrossed for >10 generations on the C57BL6/OlaHsd background that lacks alpha-synuclein (42), were after embryo transfer bred onto the C57BL6/NCrl background and then intercrossed to establish SLC^-/-^ mice with an intact alpha-synuclein locus. Mice were kept in the Gothenburg University (EBM) SPF animal facility and bred under project license authorisation (2013/81 and 2016/10). Female mice were used throughout, aged 5-6 months in most experiments except when indicated. Five-month-old female MRL^+/+^ and MRL*^lpr/lpr^* mice (The Jackson Laboratory, Bar Harbor, ME, USA) were used. WT mice were 3-months-old C57BL6/NCrl from own breeding (SLC^+/+^).

### Immunisation

WT mice were immunised intraperitoneally twice at a 30-day interval with 1×10^9^ SRBCs (Håtunalab, Sweden). Mice were sacrificed five months after booster dose.

### Flow cytometry

Spleen single cell suspensions or RBC-depleted peripheral blood leukocytes were stained with a cocktail of monoclonal antibodies (16, 18) following standard techniques and analysed on FACSVerse™ (BD Biosciences). Data were analysed using FlowJo software (Treestar Inc.).

### Cell sorting

Spleen cells were collected from pools of 3-6 mice, enriched for B cells using CD19 MACS beads (Milltenyi Biotech) or B-cell enrichment kit (StemCellTechnologies), followed by sorting FO (B220^+^CD93^-^43^-^21^int^23^+^), GC (B220^+^CD93^-^43^-^GL7^+^95^+^) B cells, MBC1 (B220^+^CD93^-^43^-^GL7^-^CD73^+^80^+^PD-L2^+^), MBC2 (B220^+^CD93^-^43^-^GL7^-^CD73^-^80^+^PD-L2^+^) and MBC3 (B220^+^CD93^-^43^-^GL7^-^CD73^+^80^+^PD-L2^-^). Cells were sorted on Icyt Synergy or BDFACSAriaIII and the purities were >90%.

### RNA extraction and qPCR

RNA was isolated using RNeasy kit (Qiagen) and cDNA was reversely transcribed using SuperScript II (Invitrogen). *Birc5*, *Zbtb32* and *Klf5* were quantified by qPCR and normalised to the expression of *Gapdh* (ThermoFisher). Primer sequences are described in (14), and Power SYBR Green Master Mix was used in the reactions (ThermoFisher). All samples were run in triplicates on a ViiA7 system and analysed with the ViiA7 basic software (ThermoFisher).

### High-throughput sequencing of Ig genes and bioinformatic analysis

The Ig sequencing protocol we used has been described in our previous publication (18). Sequences were assigned to the corresponding samples based on the MID tags. After passing the quality control criteria, sequences were submitted to IGMT/HighV-QUEST database for analysis (43). The files from IMGT/HighV-QUEST were imported into IgAT immunoglobulin analysis tool for further analysis (44). Only unique sequences were analysed. H-CDR3 and cluster analysis was carried out by the Bioinformatics Core Facility at the University of Gothenburg. For the H-CDR3 heatmap, H-CDR3 amino acid overlaps were calculated using the CalcPairwiseDistances module available from VDJtools version 1.2.1 (45). Relative overlaps were defined as the number of overlapping H-CDR3 amino acid variants divided by the total number of unique H-CDR3 amino acid variants in each pairwise sample comparison. The heatmap was constructed using ggplot2 version 3.3.5 (46). For lineage trees, the output from IMGT/HighV-QUEST was processed using Change-O version 1.2.0 (47), reads parsed with MakeDb.py and germlines reconstructed with CreateGermlines.py. Reads with shared *IGHV*, *IGHJ* genes and H-CDR3 junction region length were grouped into clones using DefineClones.py. Nucleotide hamming distance of 0.16 was used and detected using the distance to nearest neighbour module available from the R package shazam version 1.1.0 (47). Vertical phylogenetic lineages were constructed using the dnapars build available from the Phylip package version 3.695 (48). Accessed through the buildPhylipLineage module from the R package alakazam version 1.2.0 (47). Lineage trees visualised with R package ggraph version 2.0.6 (https://cran.r-project.org/web/packages/ggraph/index.html). The sequencing data are deposited in NCBI Sequence Read Archive (SRA) under the BioProject PRJNA277306 (accession code: SRP055855).

### Statistics

Mean values and standard error of the mean are displayed in the plots. P-values were calculated using appropriate statistical test: unpaired t-test or χ^2^ test (GraphPad Prism). Tukey’s test was used for the multiple comparisons. *, p<0.05; **, p<0.01; ***, p<0.001; ****, p<0.0001.

## Data availability statement

The datasets presented in this study can be found in online repositories. The names of the repository/repositories and accession number(s) can be found below: PRJNA277306 (SRA).

## Ethics statement

The animal study was reviewed and approved by Animal ethics committee of Gothenburg. Written informed consent was obtained from the owners for the participation of their animals in this study.

## Author contributions

AA, OG, and I-LM designed the experiments. AA, AC, NG, OG, and SA carried out or contributed essential reagents and materials for the experiments. AA, EE, I-LM, NG, and OG analysed and interpreted the data. UY contributed substantially to the discussions. AA, I-LM, and OG wrote the manuscript with contributions from the co-authors. All authors contributed to the article and approved the submitted version.
