# Supplementary material for: Clonal relationships of memory B cell subsets in autoimmune mice
Source: Front Immunol. 2023 Mar 1;14:1129234. doi: 10.3389/fimmu.2023.1129234 (PMC10015592; doi:10.3389/fimmu.2023.1129234)

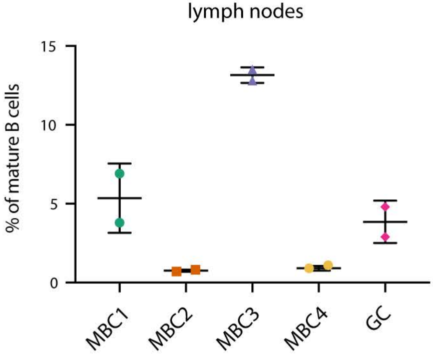

Supporting Information 1. Percentages of MBC subsets and GC B cells of mature B cells in lymph nodes from SLC$^{-/-}$ mice (n=2). Mice were 8-mo old.

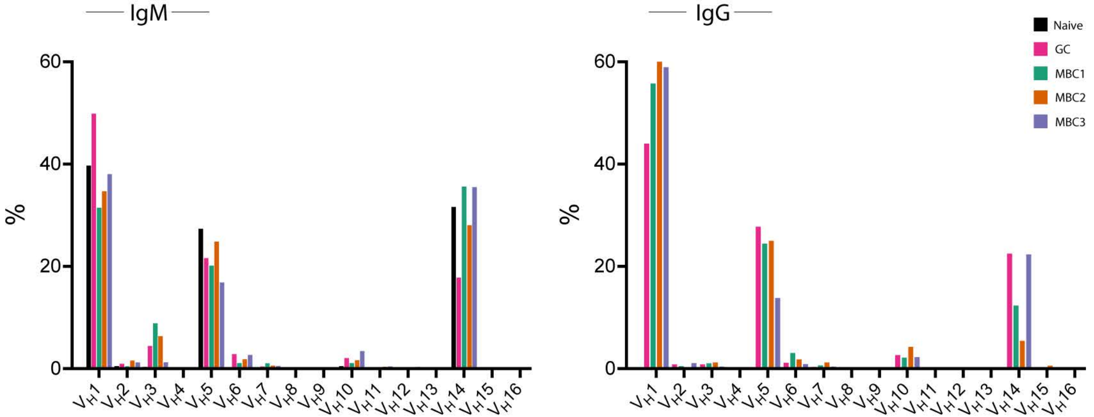

Supporting Information 2. *IGHV* family expression in naive and GC B cells as well as MBC subsets from SLC$^{-/-}$ mice.

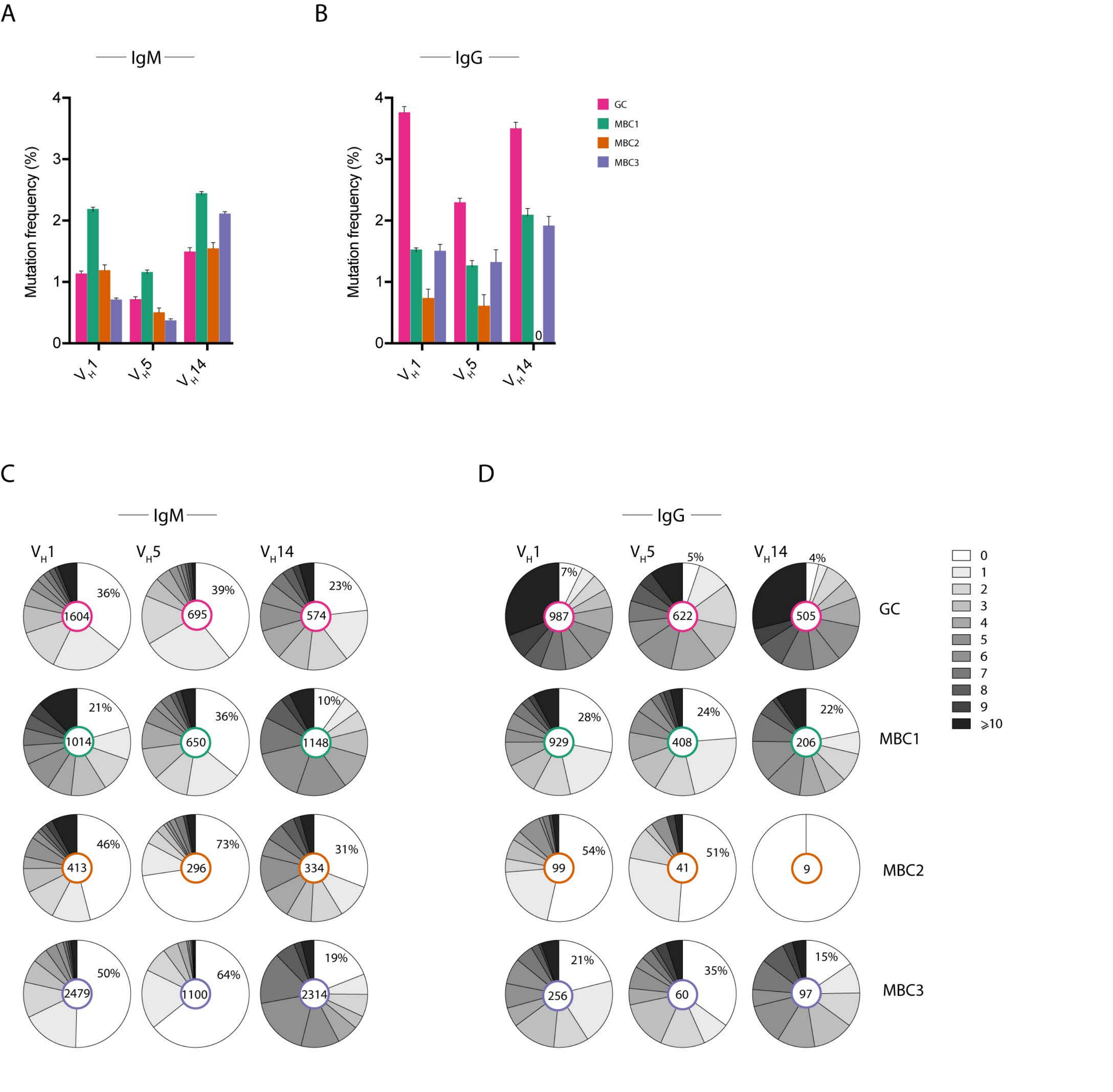

Supporting Information 3. Mutation frequency in different *IGHV* families in GC B cells and MBC subsets of IgM (A) or IgG (B) isotype from SLC$^{-/-}$ mice . (C-D) Mutation range analysis as in (A-B).

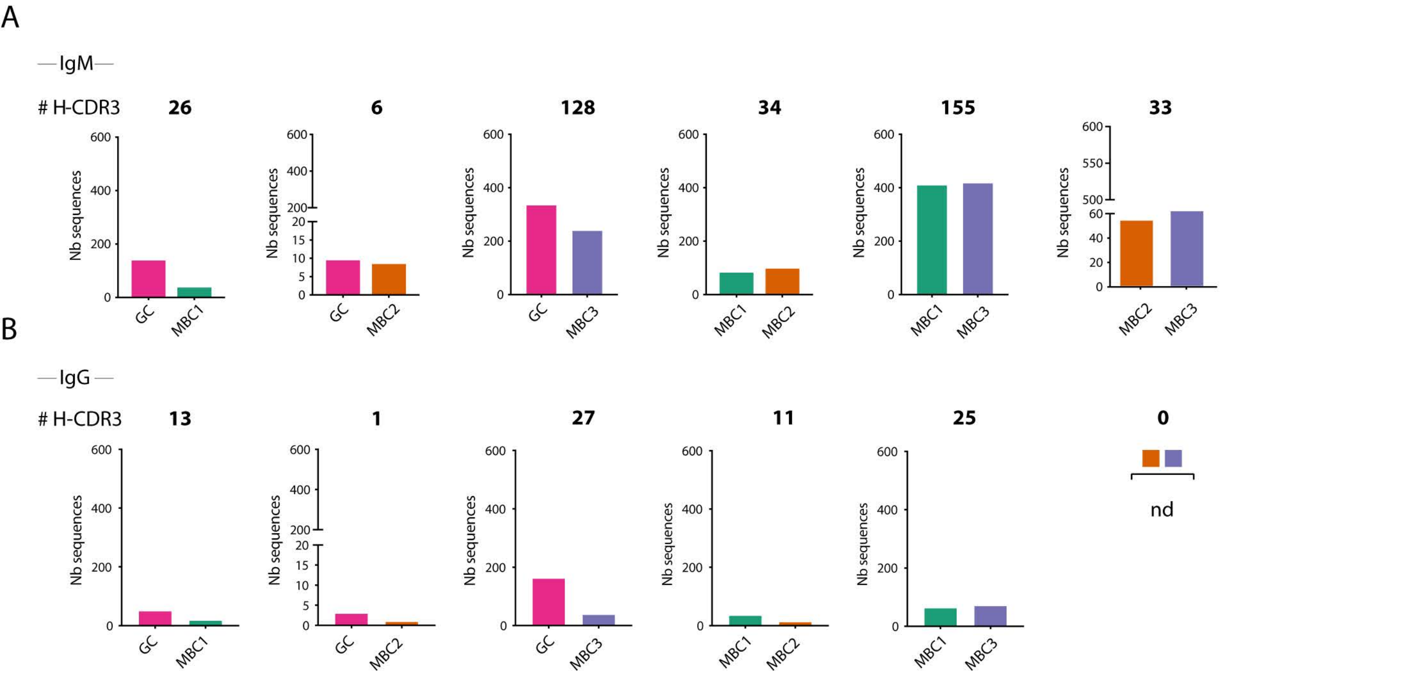

Supporting Information 4. H-CDR3 sequence overlap between GC B cells and MBC subsets of IgM (A) or IgG (B) isotype in $SLC^{-/-}$ mice. nd: not determined

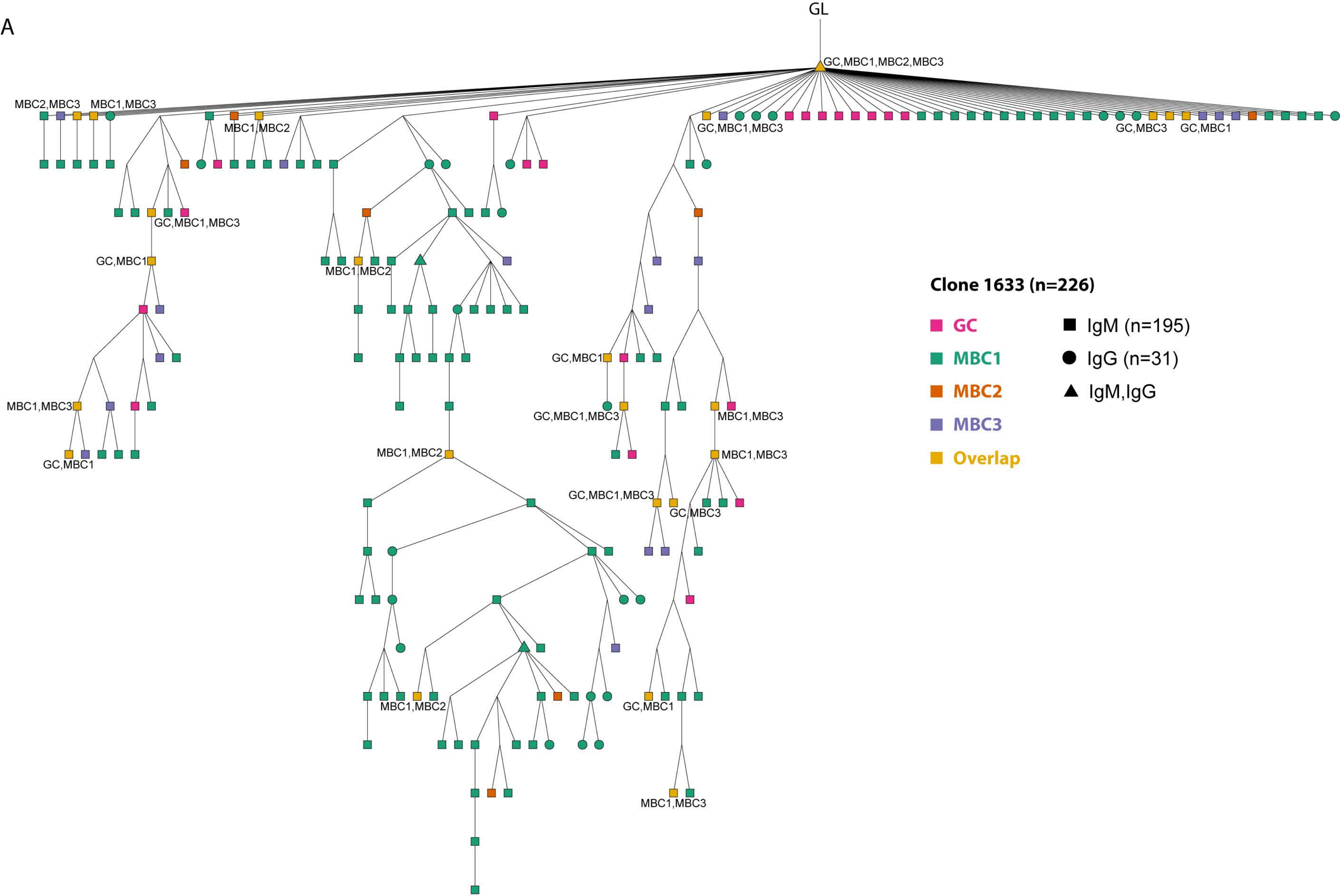

Supporting Information 5. (A-F) Lineage tree analyses showing clonal relationships between GC B cells and MBC subsets from SLC$^{-/-}$ mice. Nodes without text should be referred to the closest ancestor node.

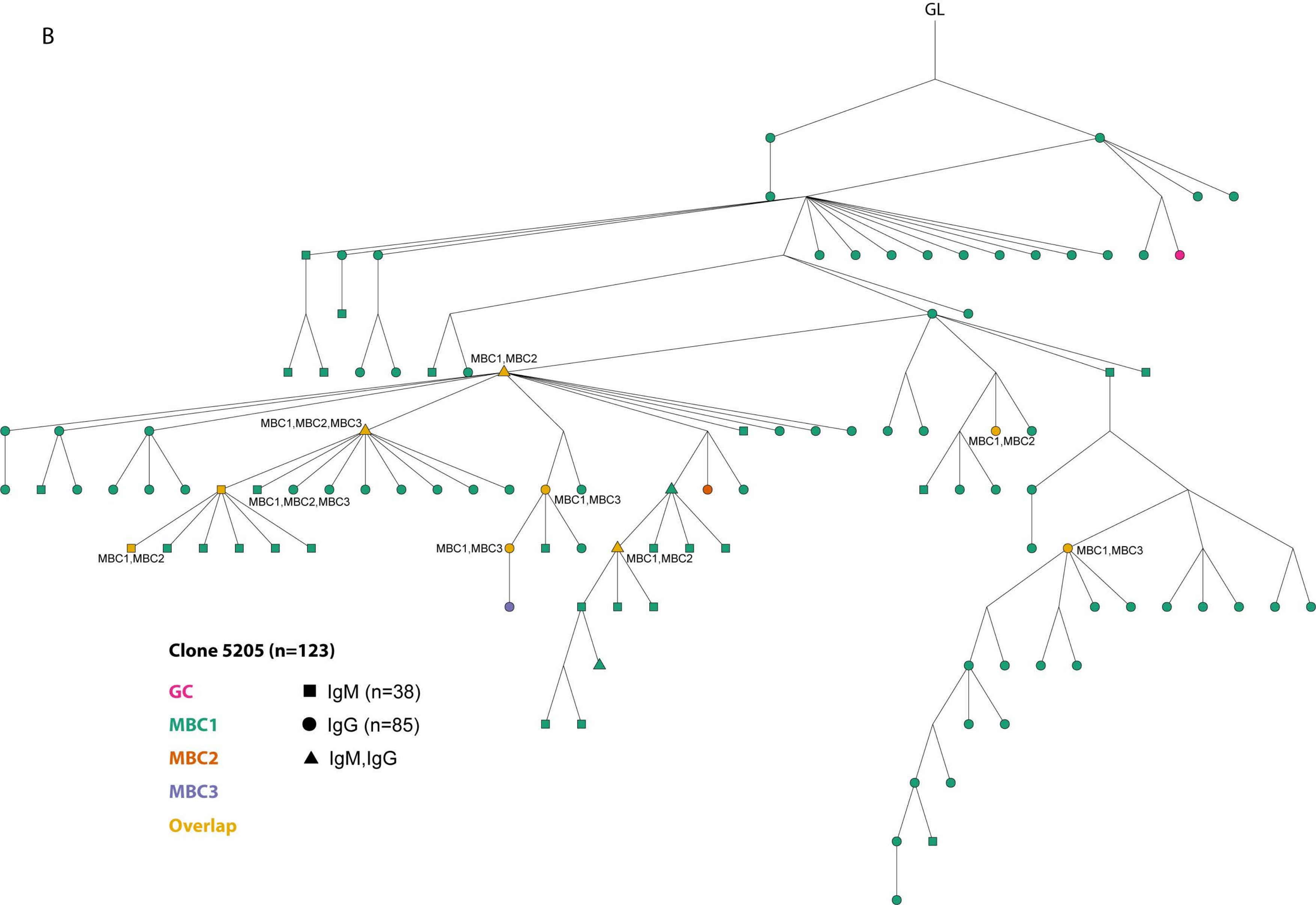
B
GL
MBC1,MBC2
MBC1,MBC2,MBC3
MBC1,MBC2
MBC1,MBC2,MBC3
MBC1,MBC3
MBC1,MBC3
MBC1,MBC2
MBC1,MBC2
MBC1,MBC3
Clone 5205 (n=123)
GC
MBC1
MBC2
MBC3
Overlap
IgM (n=38)
IgG (n=85)
IgM,IgG

C

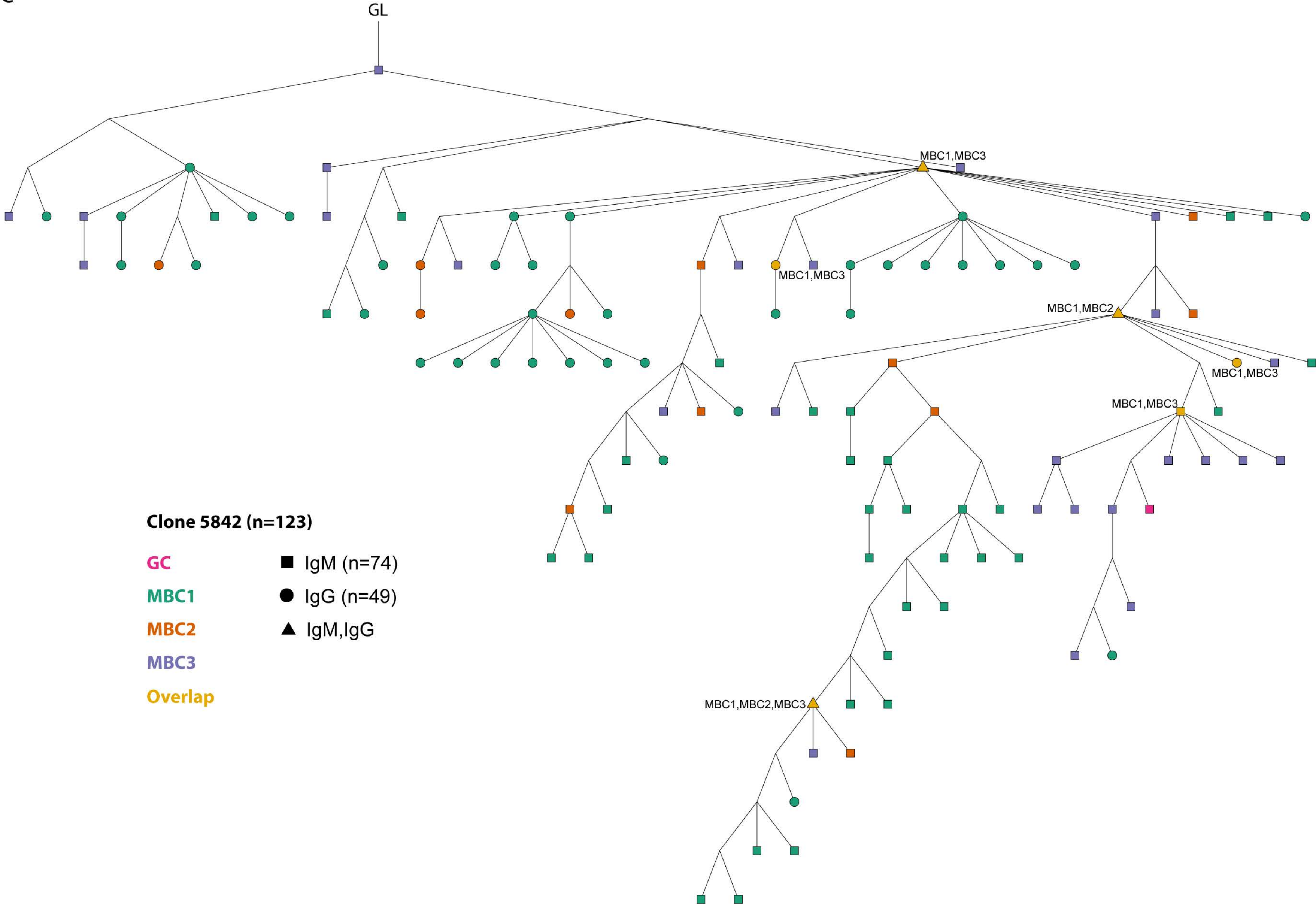
GL
MBC1,MBC3
MBC1,MBC3
MBC1,MBC2
MBC1,MBC3
MBC1,MBC3
MBC1,MBC2,MBC3
Clone 5842 (n=123)
GC
MBC1
MBC2
MBC3
Overlap
IgM (n=74)
IgG (n=49)
IgM,IgG

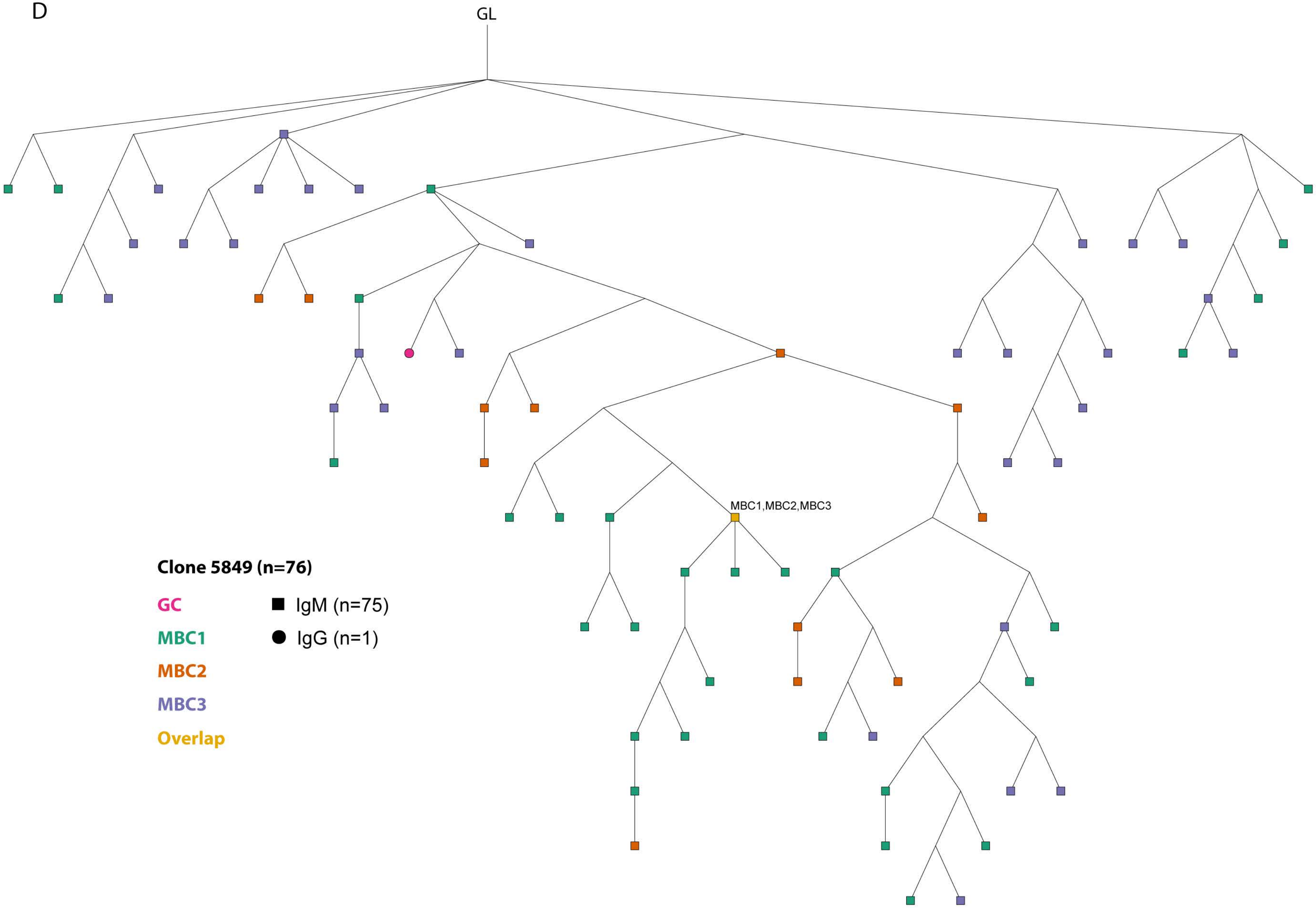
D
GL
MBC1,MBC2,MBC3
Clone 5849 (n=76)
GC
MBC1
MBC2
MBC3
Overlap
IgM (n=75)
IgG (n=1)

E

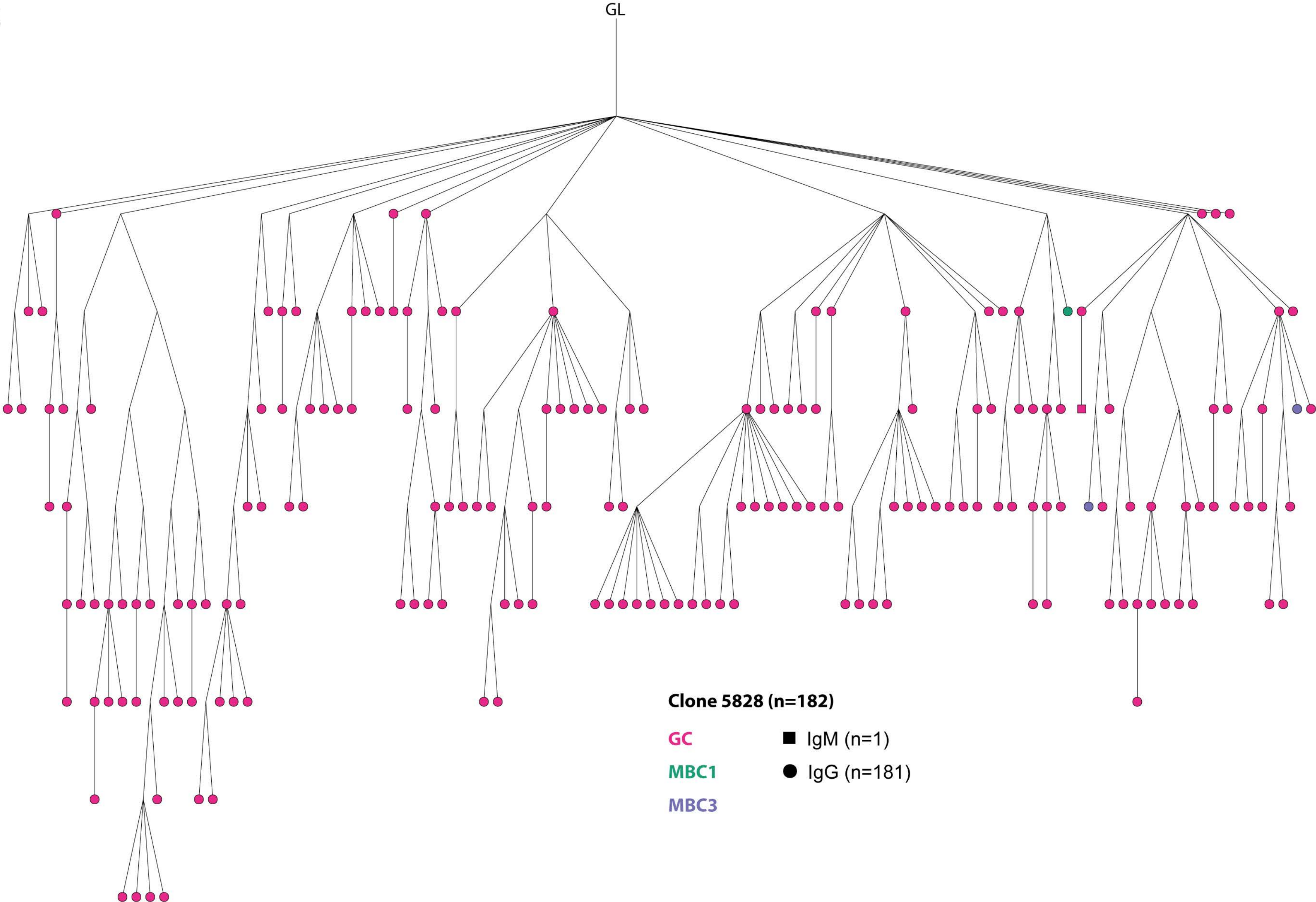

F

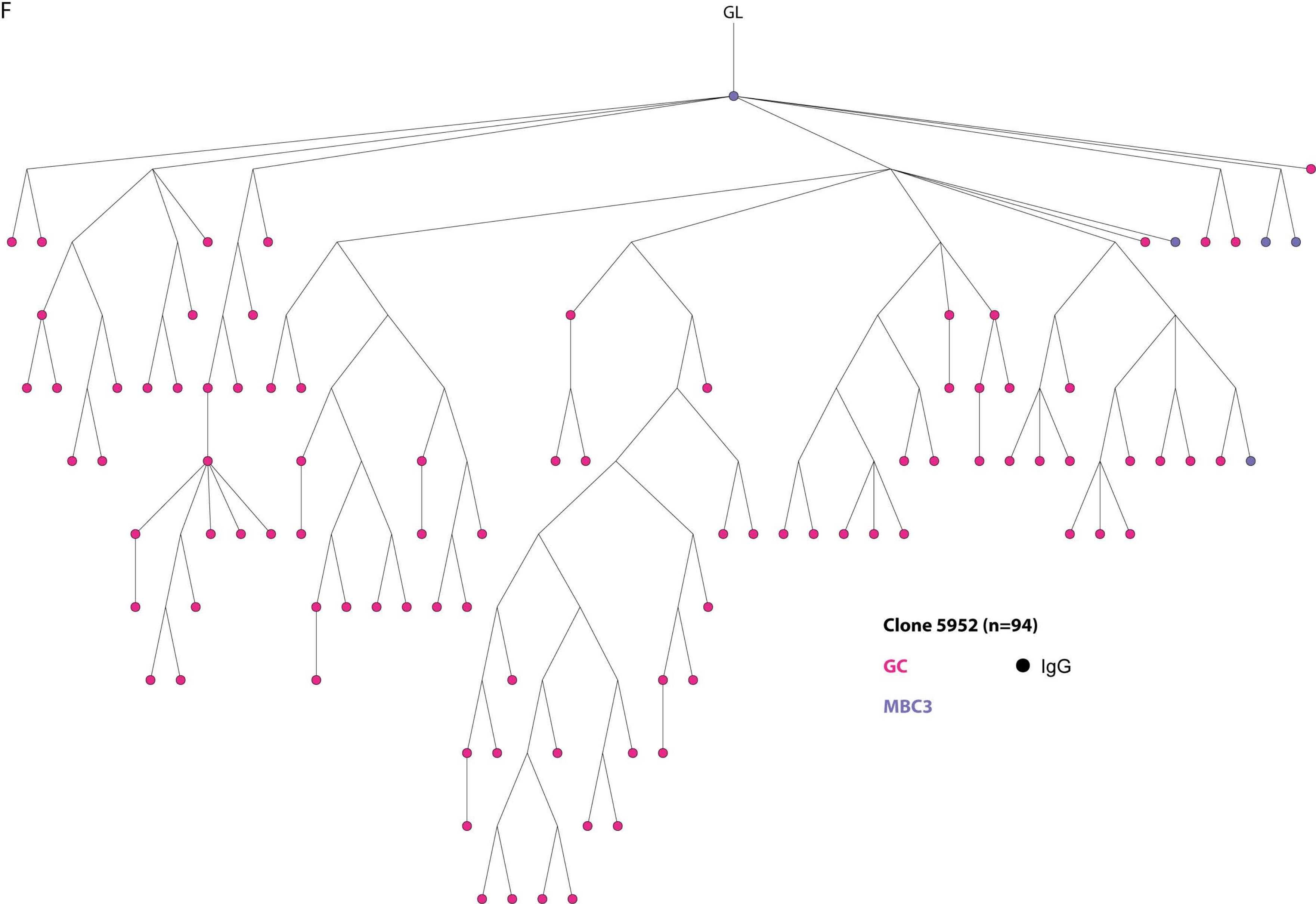

Supplement: Supplementary file 1 [file DataSheet_1.pdf]
